# A Rare Case of an Adrenal Schwannoma

**DOI:** 10.7759/cureus.33710

**Published:** 2023-01-12

**Authors:** Madiha Ahmed, Taaha Mendha, Laura J Cui, Steve Carlan, Raymond J Leveille

**Affiliations:** 1 Internal Medicine, Orlando Regional Medical Center, Orlando, USA; 2 Internal Medicine, University of Miami Miller School of Medicine, Miami, USA; 3 Internal Medicine, Florida Atlantic University Charles E. Schmidt College of Medicine, Boca Raton, USA; 4 Obstetrics, Orlando Regional Medical Center, Orlando, USA; 5 Urology, Bethesda Hospital East, Boynton Beach, USA

**Keywords:** retroperitoneal tumor, retroperitoneal laparoscopy, distal ureteral stone, adrenal disease, adrenal schwannoma

## Abstract

Schwannomas are tumors of neoplastic Schwann cells generally found in peripheral nerves in the head, neck, and extremities. They do not demonstrate hormonal abnormalities, and initial symptoms are typically secondary to adjacent organ compression. These tumors are rarely found in the retroperitoneum. We present a rare finding of an adrenal schwannoma in a 75-year-old female who presented to the emergency department with right flank pain. Imaging incidentally demonstrated a 4.8 cm left adrenal mass. Ultimately, she underwent a left robotic adrenalectomy, and immunohistochemical testing confirmed the presence of an adrenal schwannoma. It is imperative to undergo adrenalectomy and immunohistochemical testing to confirm the diagnosis and rule out malignancy.

## Introduction

An adrenal incidentaloma is an unexpected tumor on the adrenal gland discovered by imaging during an investigation for conditions not thought to be adrenal pathology. Adrenal incidentalomas are at increased risk of indicating malignancies when they are larger than 4 cm in diameter, appear bilaterally, have decreased washout on magnetic resonance imaging (MRI) or computed tomography (CT), and demonstrate irregular borders on imaging. While there are numerous differentials associated with some of these characteristics, adrenal schwannomas can be considered. Adrenal schwannomas are extremely rare and difficult to diagnose. Schwannomas are more commonly described in other parts of the body including the head, neck, and extremities [1]. It is unusual to find them in the retroperitoneum. Adrenal schwannomas make up less than 1% of all adrenal tumors. The majority of adrenal schwannomas are asymptomatic. Over the past several years, increases in imaging have incidentally led to increased detection of adrenal tumors. However, the diagnosis of an adrenal schwannoma can only be confirmed through immunohistochemical staining [2]. We present a 75-year-old female with flank pain who was incidentally found to have an adrenal mass that was eventually diagnosed to be an adrenal schwannoma after resection and immunohistochemical staining.

## Case presentation

A 75-year-old female with a past medical history of hypertension, hyperlipidemia, and gout presented to an outside facility with a chief complaint of right flank pain that started two weeks ago. She described the pain to be a moderate aching pain radiating to the right groin. CT imaging was performed and showed a 9 mm stone in the right ureter and an incidental left adrenal mass measuring 4.8 cm with no evidence of washout. The patient subsequently underwent ureteral stent placement for ureterolithiasis and was then referred to our facility for further work-up of the left adrenal mass. Upon evaluation at our facility, she admitted to having residual intermittent gross hematuria, dysuria, and right flank pain since her stent placement. A review of systems was negative. She was afebrile, and her vital signs were unremarkable. Her physical exam findings were benign including no purple striae, buffalo hump, or other cushingoid features. The pain had gradually decreased and disappeared after the stent was removed.

**Figure 1:**
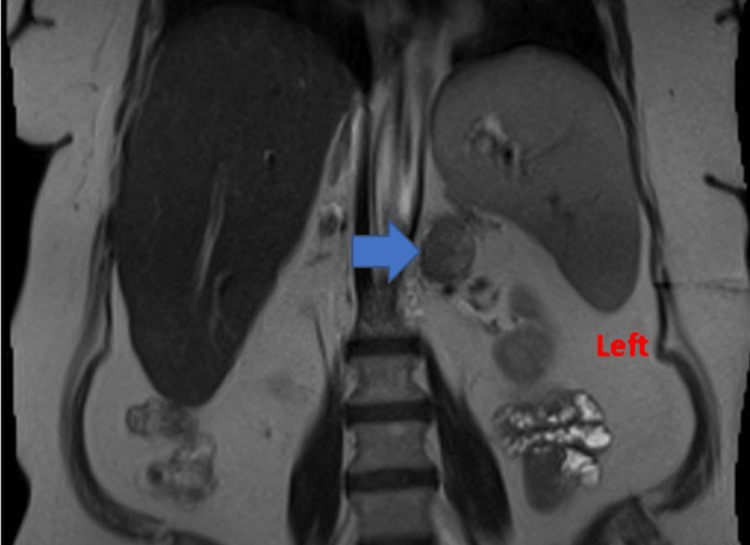
Magnetic resonance imaging of the abdomen depicting the left adrenal mass (blue arrow)

MRI of the abdomen with and without contrast was ordered and was significant for a 2.9 cm x 2.5 cm x 4.8 cm left adrenal mass, as shown in Figure 1.

There was no evidence of washout on contrast-enhanced phases. Tests for serum electrolytes, serum aldosterone to renin ratio, a 24-hour urine collection for catecholamines and metanephrines, and a low-dose dexamethasone test were performed and unremarkable.

Due to the size of the mass being over 4cm in diameter and the lack of contrast washout, there was a concern for a malignant adrenal mass or metastatic disease. She was hospitalized for a robotic left adrenalectomy, which was performed without complications. The histopathology of the specimen identified a spindle cell tumor which was close to histologically unremarkable adrenal gland tissue. Spindle cells showed no necrosis and no increased mitotic activity. Segments of nerves and ganglion cells were noted adjacent to the tumor mass, and some of the tumor cells showed nuclear pleomorphism. Immunohistochemical staining performed on the adrenal mass showed S100 protein positivity and negativity for smooth muscle actin antibody, desmin, anticytokeratin monoclonal antibodies (AE1 and AE3), synaptophysin, chromogranin, and Melan A. The patient was discharged from the hospital two days later and she continued with urology for outpatient follow-up.

## Discussion

Adrenal schwannomas are an extremely rare form of an adrenal tumor, and very few cases have been documented in the medical literature. This case highlights the significance of considering this diagnosis when assessing an adrenal mass as well as adding to the medical literature of such cases.

Adrenal incidentalomas larger than 4 cm with no evidence of MRI washout, as seen with our patient, are at increased risk of malignancy [3,4]. This case report demonstrates the importance of further investigations, such as resection and immunohistochemical staining, in establishing a diagnosis.

Our patient presented with a 4.8 cm left adrenal incidentaloma. MRI with and without contrast demonstrated a well-circumcised mass without washout on CT scans or MRI. Labs for serum electrolytes, serum aldosterone to renin ratio, a 24-hour urine collection for catecholamines and metanephrines, and a low-dose dexamethasone test were within the normal range. Immunohistochemical staining was positive for S100 and negative for smooth muscle actin, desmin, AE1/3, synaptophysin, chromogranin, and Melan A, indicating a potential diagnosis for a schwannoma.

A schwannoma is an encapsulated, benign, slow-growing neoplasm that generally occurs between the third and sixth decades of life. The main component of schwannomas arises from neural crest cells. Therefore, with the exception of cranial nerves I and II (which lack Schwann cells), schwannomas can occur in every other organ or nerve trunk [1]. These neoplasms most commonly occur in the peripheral, motor, sensory, sympathetic, or cranial nerves of the head, neck, and extremities [5,6]. They are infrequently in the retroperitoneum, accounting for approximately 1-5% of the masses in the region [7]. Moreover, they are even rarer in the adrenal region comprising only 0.7% of all adrenal tumors [8]. Five to eighteen percent of malignant schwannomas are associated with neurofibromatosis type I and II. In these diseases, they manifest in a multitude of locations [9]. 

The general patient population with a schwannoma tends to be asymptomatic. One case series of 33 cases demonstrated the median age at the time of diagnosis to be 49 years and with a female predominance. This case series also highlighted that only 13 patients were symptomatic. The other schwannomas were found incidentally [2]. The retroperitoneum is a large and malleable place, allowing for significant growth of the mass prior to the development of symptoms. Although the majority of patients remain asymptomatic, the initial symptoms are secondary to adjacent organ compression, such as vague abdominal pain, flank pain, or discomfort. A more recent case series of 31 patients indicated that only five patients were symptomatic, making 84% of the findings incidental [8]. Our patient seemed to be experiencing symptoms from her right-sided ureterolithiasis, and her contralateral adrenal schwannoma was asymptomatic.

Due to their nonfunctional status, most patients with adrenal schwannomas do not exhibit biochemical or clinical evidence of hormonal abnormalities. Nevertheless, concern for malignancy due to size and lack of imaging washout required labs to rule out the presence of other adrenal masses such as pheochromocytoma, myelolipomas, neuroblastomas, ganglioneuroma, cysts, and metastatic disease [2]. Labs for serum electrolytes, serum aldosterone to renin ratio, a 24-hour urine collection for catecholamines and metanephrines, and a low-dose dexamethasone suppression test were unremarkable, ruling out pheochromocytoma. Imaging indicated a well-circumscribed mass without macroscopic fat, ruling out myelolipomas [10]. The lack of evidence of washout on CT scans or MRI severely decreases the likelihood of a benign adenoma [4].

On CT scans, schwannomas generally appear as a round or oval mass, which is well-demarcated. There have been cases where imaging has demonstrated cystic degeneration and/or calcifications [11]. On MRI, they present as a well-circumscribed mass with low signal intensity on T1-weighted images and heterogenous high signal intensity on T2-weighted images, such as in our case [12]. Additionally, almost all schwannomas demonstrate positive immunohistochemical staining for the S100 protein as they are tumors of neuroectodermal origin [2]. However, it may be difficult to differentiate a tumor as schwannoma versus neurofibroma due to the overlap in positive S-100 protein staining. Typically, neurofibromas present cutaneously while schwannomas have been portrayed to have a positive stain for calretinin which can histologically distinguish between the two [1,13]. The absence of cutaneous lesions in our patient decreases the diagnosis of neurofibroma.

A schwannoma cannot be diagnosed until it is surgically removed. The general approach is laparoscopic, similar to the one we utilized in this case. The removal is also therapeutic as schwannomas are not responsive to either radiotherapy or chemotherapy [14]. Only subsequent immunohistochemical testing, using the methods we discussed above, can provide us the confirmatory diagnosis of the schwannoma. Post-surgical prognosis is generally positive, with a case series by Zhou et al. reporting survival without evidence of recurrence or metastasis in the postoperative time period of 7-115 months [8]. The patient continues to be well at four months since her adrenalectomy.

## Conclusions

In conclusion, adrenal schwannomas are rare tumors that are mostly found incidentally. They are difficult to diagnose and although the workup can confirm that the mass is a non-secreting adrenal mass, it is usually non-diagnostic. As confirmed by the literature and by our experience, prompt surgical excision followed by histological and immunohistochemical evaluation of the mass is crucial for accurate diagnosis and to predict the outcome. Hence, adrenal schwannomas should always be included in the differential diagnosis of solid nonfunctioning adrenal tumors.
